# Balance Impairments after Brachial Plexus Injury as Assessed through Clinical and Posturographic Evaluation

**DOI:** 10.3389/fnhum.2015.00715

**Published:** 2016-01-25

**Authors:** Lidiane Souza, Thiago Lemos, Débora C. Silva, José M. de Oliveira, José F. Guedes Corrêa, Paulo L. Tavares, Laura A. Oliveira, Erika C. Rodrigues, Claudia D. Vargas

**Affiliations:** ^1^Laboratório de Neurobiologia II, Instituto de Biofísica Carlos Chagas Filho, Universidade Federal do Rio de JaneiroRio de Janeiro, Brazil; ^2^Núcleo de Pesquisa em Neurociência e Reabilitação, Instituto de Neurologia Deolindo Couto, Universidade Federal do Rio de JaneiroRio de Janeiro, Brazil; ^3^Programa de Pós-Graduação em Ciências da Reabilitação, Centro Universitário Augusto MottaRio de Janeiro, Brazil; ^4^Instituto D’Or de Pesquisa e EnsinoRio de Janeiro, Brazil

**Keywords:** postural balance, weight-bearing, peripheral nerve injuries, brachial plexus, posturography

## Abstract

**Objective:** To investigate whether a sensorimotor deficit of the upper limb following a brachial plexus injury (BPI) affects the upright balance.

**Design:** Eleven patients with a unilateral BPI and 11 healthy subjects were recruited. The balance assessment included the Berg Balance Scale (BBS), the number of feet touches on the ground while performing a 60 s single-leg stance and posturographic assessment (eyes open and feet placed hip-width apart during a single 60 s trial). The body weight distribution (BWD) between the legs was estimated from the center of pressure (COP) lateral position. The COP variability was quantified in the anterior-posterior and lateral directions.

**Results:** BPI patients presented lower BBS scores (*p* = 0.048) and a higher frequency of feet touches during the single-leg stance (*p* = 0.042) compared with those of the healthy subjects. An asymmetric BWD toward the side opposite the affected arm was shown by 73% of BPI patients. Finally, higher COP variability was observed in BPI patients compared with healthy subjects for anterior-posterior (*p* = 0.020), but not for lateral direction (*p* = 0.818).

**Conclusions:** This study demonstrates that upper limb sensorimotor deficits following BPI affect body balance, serving as a warning for the clinical community about the need to prevent and treat the secondary outcomes of this condition.

## Introduction

The brachial plexus is formed by the spinal nerves C5 to T1 and is responsible for both motor and sensory innervation of the upper limb (Resnick, 1995). Clinical surveys indicate that 10–20% of peripheral nervous system lesions involve the brachial plexus (Narakas, 1985). Among this subset, 70% are due to auto/motorcycle traumas that generate traction forces on the neck and shoulder. Critically, there is a higher incidence of brachial plexus injury (BPI) in males younger than 30 years of age (Dubuissson and Kilne, 2002). More commonly, BPI compromises the entire brachial plexus (complete injury) or the upper trunk only (a particular case of incomplete injury; Dubuissson and Kilne, 2002; Moran et al., 2005). Remarkably, this pattern of injury makes the proximal shoulder and elbow flexors muscle groups more prone to paralysis and sensory losses, as their innervation comes from the C5-C6 (upper trunk) nerve roots (Özkan and Aydin, 2001).

The available treatments for BPI include surgical interventions, such as graft or nervous transfer (for review, see Bengtson et al., 2008) and physical therapy to rehabilitate the upper limb function (Kinlaw, 2005). However, secondary functional losses associated with BPI apart from upper limb impairment are usually not taken into consideration during physical therapy interventions. More importantly, the potential deficits in postural control and balance in these individuals have not been addressed extensively in clinical research and practice.

Previous reports have shown the contribution of the upper limb to human postural control. Studies employing axillary nerve blockage with anesthetics (Kjaergård et al., 1984), upper limb immobilization (Coleman and Clifft, 2010; Lui et al., 2013) or fatiguing arm exercises (Douris et al., 2011) have shown that decreases in upper limb function negatively affect postural control and balance, as observed by increases in body sway or reduced performance in dynamic balance tests. Altered postural control and body alignment were also found in individuals who have undergone radical mastectomy, which usually results in lymphatic edemas, restriction of movement and strength loss in the arms (Rostkowska et al., 2006; Ciesla and Polom, 2010). These findings reinforce the importance of upper limb function for adequate postural control and balance. Recently, Ridgway et al. (2013) investigated postural control deficits in children with unresolved birth BPI. The majority of the children investigated (31 out of 32) showed some sign of postural deficits, ranging from asymmetrical posture to decreased trunk rotation and atypical movements.

Despite the relevance of these studies to the understanding of the role of upper limbs in postural control, there is still no direct evidence that BPI and subsequent upper limb impairment affect postural control and balance in adults. The aim of the present study is to investigate whether postural control and balance are impaired in individuals with partial or complete BPI. Our hypothesis is that body stability in these individuals is negatively affected by upper limb dysfunction associated with this lesion.

## Materials and Methods

### Participants

Patients with BPI were recruited from the local medical and physical therapy facility from December 2011 to November 2013. The following inclusion criteria were applied: age between 18 to 40 years and a traumatic and unilateral injury involving the upper trunk or the entire brachial plexus. BPI was confirmed via surgical screening, electrodiagnostic test and clinical evaluation. Individuals were excluded if they showed other neurological injuries, Mini Mental State Exam score below 24 (Folstein et al., 1975), a history of vertigo and visual loss or uncorrected visual impairment. From an initial group of 16 subjects with BPI, eleven individuals (seven males) matched the pre-established criteria. The BPI group exhibited the following characteristics (median (range)): 29 (21–35) years of age; 174 (154–180) cm in height; and 67 (58–83) kg in weight. A control group of eleven healthy individuals matched in gender, age and corporal characteristics (27 (22–40) years of age, 176 (155–185) cm in height and 70 (64–87) kg in weight) was also recruited for the study. Anthropometrical measures and age were similar between the groups (Mann-Whitney test *p* > 0.3 for all comparisons). The experimental procedure consisted of a brief anamnesis, anthropometric measures, clinical balance tests and posturographic evaluation. All participants were fully informed about the experiment procedures and provided a written consent form. The procedures were approved by the local ethics committee (process number: 275.945).

### Balance Assessments

The Berg Balance Scale (BBS) scores and the performance during single-leg stance were employed as clinical assessments of balance in both groups. The BBS, created by Berg et al. (1989) and validated for Portuguese by Miyamoto et al. (2004), evaluates static and dynamic balance. It consists of 14 items, each one with five alternatives ranging from 0–4, with a maximum score of 56 points indicating normal balance. The balance performance on the right and left single-leg stance was quantified as the number of touches with the contralateral limb on the ground during a single-leg stance of 60 s. If and when touches on the ground occurred, the participants were instructed to return to the starting position as quickly as possible.

### Posturographic Analysis

Participants stood barefoot on a force platform (AccuSway^PLUS^, AMTI) placed 1.30 m away from the wall, with their arms relaxed next to the body, looking ahead at a marker positioned at eye level. The feet were positioned hip-width apart and parallel to the sagittal plane. The instruction was to stand quietly for 60 s. The center of pressure (COP) displacement was estimated from the ground reaction forces and moments acquired through the force platform. Data were digitized at 50 Hz, 5 Hz low-pass filtered, and stored for further analyses.

Body weight distribution (BWD) over the left and right legs was estimated based on the average COP lateral position relative to the center of the individuals’ base of support. The percentage of BWD over each limb was estimated according to the method proposed by Genthon et al. (2008), using the following equation: BWD (%) = 0.5 × COP_LAT_ + 50; with COP_LAT_ referring to COP lateral position in millimeters. This measure provides information about the asymmetrical loading of body weight on each leg: values below 50 refer to a loaded left limb; values equal to 50 mean that the body weight is equally distributed between the limbs; and values above 50 refer to a loaded right limb.

Postural sway variability was assessed through the directional stability (DS) index (Riley et al., 1995), which estimates the amount of fluctuation in the COP position and velocity in a particular direction (anterior-posterior or lateral). The DS value was calculated as the square root of the summed variances of COP displacement and velocity measured for the whole 60 s trial, separately for anterior-posterior (DS_AP_) and lateral directions (DS_LAT_).

#### Statistical Analysis

Most of the variables had non-normal distribution (Kolmogorov-Smirnov calculated *p* < 0.01). Thus, the Mann-Whitney test for independent measures was applied to check for differences in the BBS scores and COP variability (DS_AP_ and DS_LAT_) between control and BPI groups. Differences in the distribution of touches on the ground during the single-leg stance in both groups were assessed using the Chi-squared (χ^2^) test. The same procedure was applied to check for differences in the BWD between control and BPI groups. The threshold for statistical significance was set at *p* ≤ 0.05.

## Results

### Participants

The clinical data of the selected individuals are presented in Table 1. From the eleven individuals included in the BPI group, five had a complete (C5-T1), and six had a partial (C5-C6 or C5-C7) BPI lesion. The time elapsed from lesion to inclusion in the experiment was median (range) 14 (2–59) months. Eight individuals with BPI underwent surgical reconstruction of the brachial plexus, and three did not undergo any surgical procedure. None of the individuals in the BPI group exhibited considerable muscle strength in the biceps brachii muscle (MRC scale ≥ 3).

**Table 1:** **Individual characteristics of patients with brachial plexus injury (BPI)**.

Patient ID	Gender	Age	Lesion time	Injured side	Biceps force	Diagnosis	Surgical procedure
P01	M	30	2	Left	0	C5-C8, T1	None
P02	F	23	2	Left	2	C5-C6	None
P03	M	21	13	Left	0	C5-C8, T1	None
P04	F	26	20	Right	2	C5-C7	NG/NT
P05	M	32	59	Right	0	C5-C8, T1	NT
P06	M	21	14	Right	1	C5-C8, T1	NG
P07	F	35	19	Left	0	C5-C8, T1	NT
P08	F	34	53	Right	0	C5-C7	NT
P09	M	34	7	Right	1	C5-C7	NT
P10	M	20	11	Left	0	C5-C6	NT
P11	M	35	55	Right	0	C5-C6	NT

*Age in years; Lesion time in months; Biceps brachii muscle force graded according to Medical Research Council (MRC) scale; NG, nerve graft; NT, nerve transfer*.

### Balance Assessment

There was significant difference in the static and dynamic balance measures between the groups. The BPI group scored lower in BBS than did the control group (*z* = −1.98, *p* = 0.048; Table 2). Additionally, a significant difference in the single-leg stance performance was observed between the groups (χ^2^ = 4.12, *p* = 0.042). The BPI group showed a higher frequency of feet touches on the platform compared with that of the control group (Table 2): six BPI individuals touched the platform 1–5 times (a total of 15 touches), while three healthy individuals touched the platform once (a total of three touches).

**Table 2:** **Clinical balance measures**.

	BPI group	Control group	Statistical results
Berg Balance Scale score	54 (54–56)	56 (56–56)	*z* = −1.98 *p* = 0.048
Single-leg stance performance*			
*Yes*	6	3	χ^2^ = 4.12 *p* = 0.042
*No*	5	8	
BWD**			
*Injured/Non dominant arm*	2	4	χ^2^ = 5.00 *p* = 0.025
*Non injured/Dominant arm*	8	4	

Group data from Berg Balance Scale are presented as median (lower-upper quartile). Statistical results are shown as the correspondent distribution value (z or χ^2^) and p-value. *Expressed as the number of subjects that touch the ground at least once during 60 s left or right single-leg stance. **Subjects with body weight distribution (BWD) of 50% (i.e., symmetrical BWD) were not included in the analysis; see text for further details. BPI, brachial plexus injury.

### Body Weight Distribution (BWD)

BWD differences between groups were assessed by calculating the number of subjects who distributed their weight to the side of the injured/non-injured upper limb (BPI group) or to the dominant/non-dominant upper limb (control group). It was observed that eight BPI individuals (73% of the total sample) distributed their body weight predominantly toward the side opposite to the affected arm, while two BPI individuals (18%) held their body weight predominantly on the lesion side. However, four of the control individuals supported their body weight contralateral to the dominant arm (defined by the Edinburg questionnaire) and four held their weight on the ipsilateral side. One patient and three control individuals sustained their body weight at the very center of their base of support (i.e., BWD = 50%); to simplify the interpretation, they were excluded from the statistical analysis. The BWD for the injured/non-dominant arm side vs. the non-injured/dominant arms side (for the BPI/control group, respectively) showed significant differences between groups (χ^2^ = 5.00, *p* = 0.025; Table 2).

### Postural Sway Variability

**Figure 1:**
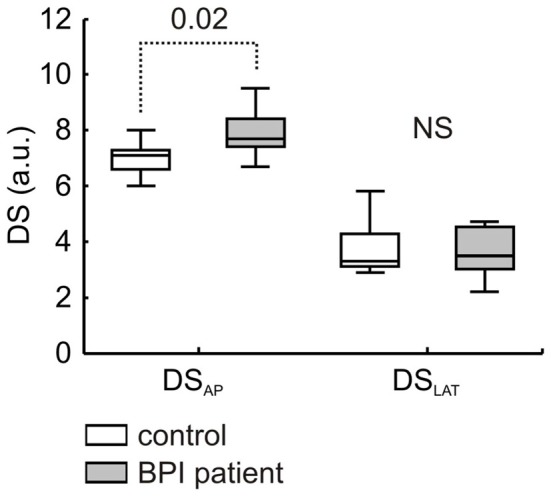
**COP directional stability values in anterior-posterior (DS_AP_) and lateral (DS_LAT_) directions.** Median, lower-upper quartiles and min-max values are represented. Data were presented for control (white bars) and patients with brachial plexus injury (gray bars) groups (*n* = 11 for each group). Dotted line refers to significant difference, and number inset refers to Mann-Whitney *p*-value. NS indicates no significant difference (*p* = 0.82).

A larger variability in COP displacement in the anterior-posterior direction was found for BPI individuals compared with that of the control group (*z* = −2.33 *p* = 0.020; Figure 1). No effect was found in the lateral direction (*z* = −0.23, *p* = 0.818).

## Discussion

In the present study, we aimed to investigate whether postural control and balance were impaired in individuals with partial or complete BPI. To achieve this goal, the postural control and balance performance of BPI individuals were compared with those of healthy individuals. The main results were that, compared with a control group paired in gender, age and anthropometric characteristics, BPI individuals: (i) had lower scores in the BBS; (ii) were more unstable during single-leg stance; (iii) distributed their body weight toward the side opposite the affected arm; and (iv) showed higher COP variability during quiet stance.

Clinical balance assessment was made through BBS and single-leg stance performance, and the results of both tests indicate that BPI individuals performed poorly under static and dynamic conditions compared with their healthy counterparts. Although the difference between groups consisted in two points of the BBS (median) score (see Table 2), this result had both statistical and clinical significance. The median score obtained for BPI individuals was comparable to that reported for sedentary elderly people (approximately 53 points Coleman and Clifft, 2010), despite an average age difference of 46 years between this sample and that of Coleman and Clifft (2010). All BPI individuals showed a severe functional impairment mainly in the proximal muscles of the upper limb (see Table 1). Interestingly, poor performance on the BBS was also found for elderly people exposed to shoulder/elbow restriction (Coleman and Clifft, 2010). In this population, upper limb immobilization promotes an average reduction of one point in the BBS score. A simple predictive model of fall status of elderly people indicates that within the upper range of BBS (near 54–56 points) each 1-point reduction is associated with 3–4% increase in the risk of a fall (Shumway-Cook et al., 1997); extending their findings to this study, BPI individuals shows an approximately 8% increase in the risk of a fall compared with their healthy counterparts.

Single-leg stance performance also indicated impairment in postural control and balance in BPI individuals. Half of the BPI individuals suffered from imbalance during the single-leg test, compared with approximately 1/3 of the control group (see Table 2). The number of touches on the ground was also five times higher in the BPI individuals compared with their healthy counterparts. It was previously demonstrated that single-leg stance performance is an adequate test to discriminate fallers from non-fallers in elderly people (MacRae et al., 1992), and this test has been recommended as a good predictor of fall risk in this population (Hurvitz et al., 2000). Although single-leg stance instability was usually related with an impairment in distal lower limb segments such as the ankle joint, this instability is accompanied by larger upper body corrections. Tropp and Odenrick (1988) and Douris et al. (2011) provided further evidence that changes in upper limb function also contribute to single-leg stance stability. Young individuals were submitted to balance evaluation, and the authors found that the single-leg stance instability doubled after performing fatiguing exercises of the upper limbs. Altogether, upper limb functional impairments significantly impact single-leg stance performance, which could predict balance disorders and an increased risk of a fall in BPI individuals.

This study also showed that BPI individuals had higher asymmetry in weight bearing to the side opposite to the affected arm, i.e., to the non-affected side. One potential explanation for this body weight asymmetric distribution is an overall postural misalignment associated with upper limb sensorimotor impairment, as already observed in children with BPI at birth (Ridgway et al., 2013) and in women submitted to mastectomy—this last condition being associated with asymmetric trunk posture and shoulder girdle alignment (Rostkowska et al., 2006; Ciesla and Polom, 2010). These apparently distinct clinical conditions share a dramatic upper limb functional impairment, and similar postural impairments should hypothetically be expected.

Together with the weight-bearing asymmetry, posturographic assessment also revealed a large COP variability in BPI individuals, particularly in the anterior-posterior direction (Figure 1). Increases in the COP displacement and velocity, measured through the DS index, were observed under several clinical (Riley et al., 1995) and experimental conditions (Lemos et al., 2014). Although not necessarily related with the postural instability (see an interesting review in Haddad et al., 2013), a large COP displacement and/or higher velocities impaired the ability to sustain a standing position, as the COP begins to approach the individual’s limits of stability (Pai and Patton, 1997). In addition, a larger COP variability could be interpreted as the level of regulatory activity (in terms of active stiffness and sensory noise) associated with postural stabilization (Maurer and Peterka, 2005). The fact that BPI individuals exhibit larger COP variability during a quiet stance position indicates altered (impoverished) postural control compared with that of healthy individuals, stressing the marked functional role of upper limb segments on overall postural control and balance.

The role of the upper limb in human postural control has previously been reported indirectly. It has been shown, for example, that axillary nerve blockage with anesthetics produces a 30% increase in body sway (Kjaergård et al., 1984), while immobilization of the upper limb with a sling reduce the performance in dynamic balance tests and increase the number of falls (Lui et al., 2013). The present study provides evidence that postural control and balance in adult subjects is impaired by upper limb dysfunction associated with the brachial plexus lesion. At least two potential mechanisms could explain the altered balance performance in BPI patients: (i) a biomechanical compensation for the lack of torque generation of the upper limb; and (ii) changes in central sensorimotor representations following BPI.

The soundest evidences linking upper limb function to whole-body biomechanics comes from the studies investigating the role of arm swinging during walking. These studies suggest that upper limbs motion (being it passively or actively-driven) act as a damper to reduce trunk and head rotation and minimize energy expenditure during normal walking (Collins et al., 2009; Pontzer et al., 2009). Interestingly, BWD and gait performance of upper limb (shoulder level) amputees was improved with the use of a functional arm prosthesis (Bertels et al., 2012), which reinforce the role of inertial properties of the arm in overall body stabilization. In what concerns BPI patients, the lack of passive (i.e., elastic) and active (muscle-driven) torque generation due to muscle and connective tissue loss could impair the proper biomechanical link between upper arm and trunk, with consequent impairments in whole-body upright position control. This hypothesis should be addressed in future studies.

Along with mechanical factors, central adaptations to peripheral nerve injury could also influence the balance response of these patients. Injury of peripheral nerve or spinal nerve roots avulsion lead to significant changes in cortical representations of the affected muscles (e.g., Cohen et al., 1991; Mercier et al., 2006). In the particular case of BPI patients, a reorganization of cortical sensorimotor representation of the injured and non-injured muscles as well as reduction in M1 interhemispheric functional connectivity have been described through neuroimaging and transcranial magnetic stimulation (TMS) approaches (Malessy et al., 1998, 2003; Liu et al., 2013). There are few evidences that sensorimotor representation of trunk and proximal muscles could affect postural control. The finest one comes from the study of Tsao et al. (2008) showing that changes in TMS-evoked cortical representation of abdominal muscles is associated with impaired postural responses in low back pain patients. Additionally, Kantak et al. (2013) showed that modifications of body posture from sitting to standing position is sufficient to increase corticospinal excitability of proximal but not distal muscles of the upper limb. However, whether and how central nervous system reorganization contributes to the observed postural control impairments of patients suffering from BPI is still a matter of debate.

## Study Limitations

One of the limitations of the current study is the heterogeneity of the investigated sample, mainly with respect to the lesion type (complete or incomplete), the occurrence of surgical intervention and the time elapsed from lesion (see Table 1). The level of residual upper limb function depends on the extent of peripheral nerve lesion and the type of lesion as well as the subsequent surgical procedure are among the main factors contributing to proper recovery (Dubuissson and Kilne, 2002; Moran et al., 2005). In addition, the broad range of time elapsed from lesion could also impact body balance performance in these patients. Specifically, peripheral nerve lesion causes continuous, time-dependent adaptation in the cortical network (Jiang et al., 2010; Qiu et al., 2014) that could potentially affect motor function. Moreover, balance control is highly adaptive, and postural sway measures could be different in acute compared to chronic impairment; for example, BWD is improved (Sackley and Lincoln, 1997) and spontaneous postural sway is reduced (Mizrahi et al., 1989) in stroke patients a few weeks after the lesion.

Irrespectively of these factors, however, all of the individuals showed impairments in the proximal arm muscles, corresponding to a lesion at the level of the brachial plexus upper trunk (Dubuissson and Kilne, 2002; Moran et al., 2005). The anatomical characteristics of proximal muscles (i.e., their size and attachment pattern at the trunk), as well as their role in distal segment stabilization (Hasan, 2005), increase the likelihood that they will influence body posture and balance. Therefore, despite differences in the individual clinical characteristics, the functional deficit that potentially affects postural control and balance is essentially the same, and it could be assumed that the observed heterogeneity had only a minor impact on the study outcome. Nevertheless, a large BPI sample is necessary to unravel the influences of lesion type and extent, as well as the effects of surgical intervention and time elapsed from lesion on postural control and balance measures in this population.

## Conclusion

Altogether, clinical balance assessment and posturographic analysis in the BPI group indicate that these individuals exhibit postural control and balance impairments. These results suggest that motor impairment after BPI is not restricted to the upper limb segment and could impact the overall functional health status of these individuals. Our findings indicate that rehabilitation after brachial plexus lesion should not be directed only to the upper limb and that interventions that aim to improve balance could be suitable for this population. Further investigations must be performed to evaluate balance intervention efficacy in BPI individuals.

## Author Contributions

LS and TL contributed equally to this work. This manuscript is authored by LS, TL, DCS, JMdO, JFGC, PLT, LAO, ECR and CDV and all the authors contributed substantially to the areas indicated: (1) substantial contributions to the conception or design of the work; the acquisition, analysis, or interpretation of data for the work; (2) drafting the work or revising it critically; (3) final approval of the version to be published; (4) agreement to be accountable for all aspects of the work in ensuring that questions related to the accuracy or integrity of any part of the work are appropriately investigated and resolved.

## Funding

This study was supported by CNPq, CAPES, FAPERJ. This research has been conducted as part of the activities of FAPESP Research, Dissemination, and Innovation Center for Neuromathematics (Grant 2013/07699-0, S. Paulo Research Foundation). LS is a recipient of a fellowship from CAPES. No commercial party having a direct financial interest in the results of the research supporting this article has conferred or will confer a benefit on the authors or on any organization with which the authors are associated.

## Conflict of Interest Statement

The authors declare that the research was conducted in the absence of any commercial or financial relationships that could be construed as a potential conflict of interest.
